# Downregulation of the Yes-Associated Protein Is Associated with Extracellular Matrix Disorders in Ascending Aortic Aneurysms

**DOI:** 10.1155/2016/6786184

**Published:** 2016-01-20

**Authors:** Haiyang Li, Wenjian Jiang, Weihong Ren, Dong Guo, Jialong Guo, Xiaolong Wang, Yuyong Liu, Feng Lan, Jie Du, Hongjia Zhang

**Affiliations:** ^1^Department of Cardiac Surgery, Beijing Anzhen Hospital, Capital Medical University, Beijing 10029, China; ^2^Beijing Institute of Heart, Lung and Blood Vessel Diseases, Beijing 10029, China; ^3^Beijing Lab for Cardiovascular Precision Medicine, Beijing 10029, China; ^4^The Key Laboratory of Remodeling-Related Cardiovascular Disease, Ministry of Education, Beijing 10029, China; ^5^Beijing Aortic Disease Center, Cardiovascular Surgery Center, Beijing 10029, China; ^6^Beijing Engineering Research Center for Vascular Prostheses, Beijing 10029, China; ^7^Beijing Collaborative Innovation Center for Cardiovascular Disorders, Beijing 10029, China

## Abstract

Previous studies indicate that extracellular matrix (ECM) disorders lead to the apoptosis of Vascular Smooth Muscle Cells (VSMCs), which impairs the aortic wall by reducing the generation of elastic fibers, and ultimately result in ascending aortic aneurysm. The critical role of the Yes-associated protein (YAP) has been elucidated in cardiac/SMC proliferation during cardiovascular development. However, the association of YAP expression and extracellular matrix disorders in ascending aortic aneurysms is not clear. Here, we present for the first time that the downregulation of YAP in VSMCs is associated with ECM disorders of the media in ascending aortic aneurysms. We found that aortic ECM deteriorated with increased apoptotic VSMCs. Moreover, expression of YAP was dramatically reduced in the aortic walls of patients with ascending aortic aneurysms, while the normal aortic samples exhibited abundant YAP in the VSMCs. These results suggest that downregulation of YAP leads to apoptosis of VSMCs, which are essential for the homeostasis of the aortic wall. The resultant ECM disorders affect aortic structure and function and contribute to the development of ascending aortic aneurysms. In summary, through assessment of clinical samples, we revealed the association between downregulation of YAP in VSMCs and the development of ascending aortic aneurysms, providing new insight into the pathogenesis of this disease.

## 1. Introduction

Ascending aortic aneurysm is a high-risk vascular disorder, which presents as a dilatation or bulging of the ascending aorta that has a variety of causes, such as damage to the aortic wall. As the ascending aortic aneurysm progresses, the weakened aortic wall is unable to withstand the pressure of blood flow and ultimately ruptures [1]. Thus, ascending aortic aneurysms are among the most fatal cardiovascular diseases. At present, both the pathogenesis and pathophysiology of ascending aortic aneurysms are not completely clear, but Vascular Smooth Muscle Cells (VSMCs) are recognized as the most important factor in the development of ascending aortic aneurysms [1, 2]. Existing studies suggest that aortic disorders are likely to occur when the aortic wall cannot handle the stress [1, 3–5]. Mechanical disruption, which is related to disorders of the extracellular matrix (ECM), plays an important role in aortic wall remodeling and induces apoptosis of VSMCs [6, 7].

The Yes-associated protein (YAP), which is involved in the regulation of cell proliferation and apoptosis, is one of the most significant cellular signaling participants* in vivo* [4, 8]. Recent studies have shown that YAP regulates the expression of genes involved in cell proliferation and cell cycle and apoptosis at the transcriptional level [8], and YAP promotes cell division and maintenance of cell life [9]. The functional role of YAP has also been implicated in cardiac/SMC proliferation during cardiovascular development [10]. According to our previous study, YAP plays a key role in hypertrophic cardiomyopathy [11]. Additionally, abnormal aorta is observed in smooth muscle-specific* YAP*-KO mice [10]. Therefore, we asked whether YAP is also involved in the extracellular matrix disorders in ascending aortic aneurysms through regulation of the functions of VSMCs. Our results showed that YAP was downregulated in human ascending aortic aneurysm samples, and increased apoptosis of VSMCs was observed. We hypothesized that the downregulation of YAP is related to damage to the ECM in ascending aortic aneurysms, which has been implicated in tumorigenesis [12, 13], and led to the increased apoptosis of VSMCs. We also revealed that the occurrence of aortic diseases such as ascending aortic aneurysms is closely related to VSMC apoptosis, which is consistent with previous studies [14–16]. In summary, our findings show that downregulated YAP is associated with ECM disorders and contributes to the development of ascending aortic aneurysms.

## 2. Materials and Methods

### 2.1. Clinical Samples

The ethics committee of our institute approved the use of human aortic tissue samples. Normal human aortic samples were obtained from heart transplantation donors. Human ascending aortic aneurysm samples were obtained from patients undergoing surgical replacement of the ascending aorta. Patients with genetic aortic diseases, such as Marfan's, Turner, Loeys-Dietz, or Ehlers-Danlos syndrome, were excluded. All tissue samples were collected immediately after they were resected during the surgery. The clinical information of the patients is shown in Table 1.

### 2.2. Elastin Staining

For elastin staining of the human aorta, an established method was performed using an elastic fiber staining kit (Maixin Bio) [17]. To remove the paraffin, sections were treated with xylene and rehydrated. Then, the following procedure was carried out: sections were incubated for 5 min in Lugol's iodine solution, washed with PBS, incubated with sodium thiosulfate for 5 min, washed with PBS and 70% ethanol, incubated with aldehyde-fuchsin for 10 min, and then incubated with acid Orange G for seconds.

### 2.3. Western Blot Analysis

Human aortic samples were harvested and stored at less than −80°C. Protein was extracted using a protein extraction kit containing protease inhibitor and Protein Phosphatase Inhibitor Cocktail. Equal amounts of protein extracts (40 *μ*g/lane) were separated with a 10% SDS-PAGE gel. The expression levels of the GAPDH and YAP proteins were probed by incubation with the primary antibodies anti-GAPDH (1 : 2000 dilution, Sigma-Aldrich) and YAP (1 : 1000 dilution, Cell Signaling), respectively, for over 6 hours at 4°C, followed by incubation with IR dye-conjugated secondary antibodies (1 : 5000, Rockland Immunochemicals, Gilbertsville, PA) for 1 hour. Then, the results were analyzed using an Odyssey infrared imaging system (LI-COR Biosciences Lincoln, NE).

### 2.4. Histology and Immunohistochemistry

Human aortic samples were fixed in 10% formalin, embedded in paraffin, and sectioned at 5 *μ*m intervals. Immunohistochemical staining was performed using established methods [17]. To remove the paraffin, sections were treated with xylene and rehydrated. Then, they were incubated with 3% H_2_O_2_ for 10 min at room temperature and washed 3 times with phosphate-buffered saline (PBS). After blocking with serum for 30 min, the sections were incubated with primary antibodies against YAP (1 : 1000 dilution, Cell Signaling), *α*- smooth muscle actin (*α*-SMA, 1 : 500 dilution, Sigma), Bcl-2 (1 : 1000 dilution, Cell Signaling), and cleaved caspase-3 (1 : 300 dilution, Cell Signaling), followed by incubation with the ChemMate EnVision System (Dako). ImageProPlus 3.0 (ECIPSE80i/90i) was used to capture the images and analyze the results. For cryostat sections, human and mouse aortic samples were fixed in 4% paraformaldehyde, embedded in optimum cutting temperature (OCT) compound, frozen in liquid nitrogen, and sectioned at 5 *μ*m intervals. DeadEnd Fluorometric TUNEL (Promega) was used to detect the apoptotic cells. Apoptotic VSMCs were detected by TUNEL and *α*-SMA double staining before confocal fluorescence microscopy analysis (Leica Microsystems).

### 2.5. Statistical Analysis

Statistical analysis of the data was performed with a two-tailed Student's *t*-test. The data are presented as mean ± SEM, with the exception of the luciferase assay data in which mean ± SD is shown. ^*∗*^ *p* < 0.05 or ^*∗∗*^ *p* < 0.01 denotes statistical significance.

## 3. Results

### 3.1. Aortic Wall Samples of Patients with Ascending Aortic Aneurysms Exhibit Damage to the ECM and Increased Apoptosis of VSMCs

**Figure 1:**
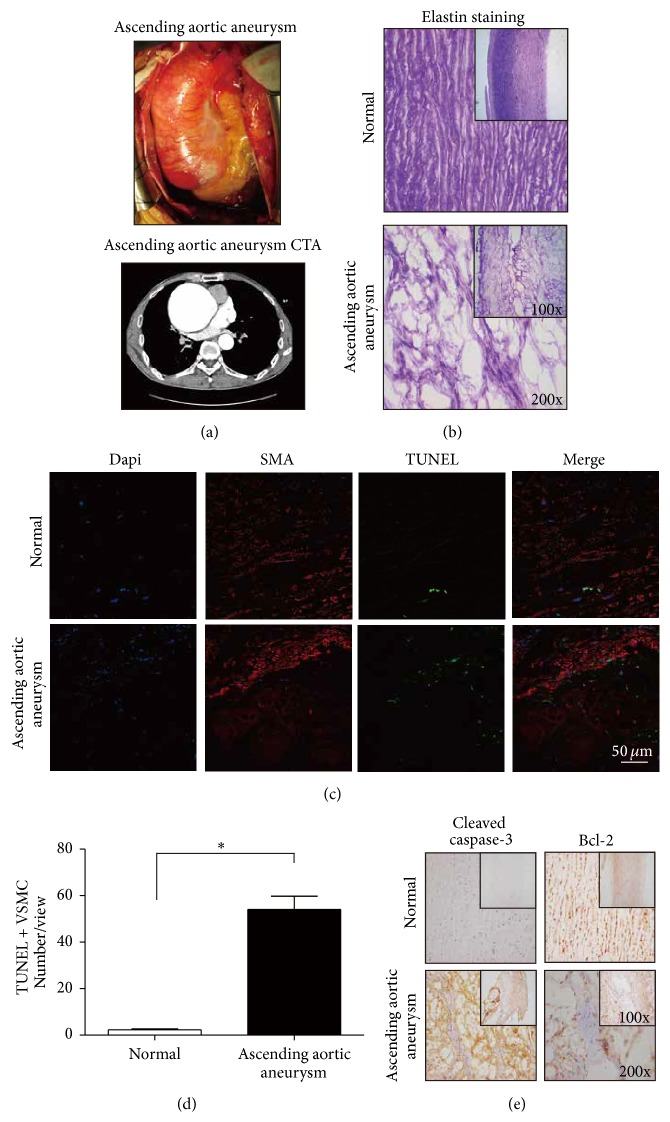
(a) Intraoperative images and CTA showing the enlarged ascending aorta in an ascending aortic aneurysm. (b) Elastin staining showed obvious derangement of tissue structure of the ascending aorta in ascending aortic aneurysm. (c, d) Confocal fluorescence microscopy showed that the number of TUNEL and *α*-SMA double-staining cells was clearly increased in the ascending aortic walls of ascending aortic aneurysms (^*∗∗*^ *p* = 0.0014). (e) Immunohistochemistry showed that the cleaved caspase-3 staining was strong and Bcl-2 staining was weak in the ascending aortic walls of ascending aortic aneurysms relative to normal.

To investigate the pathogenesis of ascending aortic aneurysms, human aortic wall samples from 8 patients undergoing ascending aorta replacement and age-matched heart transplantation donors were collected at our institute. The clinical information of the patients is shown in Table 1. There were 5 males and 3 females with a mean age of 61 ± 8.02 (range: 52 to 72) years. The mean diameter of the ascending aorta from patients with ascending aortic aneurysms calibrated by preoperative echocardiography was 55.75 ± 6.16 mm, and the mean diameter of the aortic sinus was 43.25 ± 7.89 mm. Two patients suffered simultaneously from aortic bicuspid. Various patients underwent different surgeries such as the Bentall procedure and ascending aorta replacement sufficient for the level of aortic involvement, as well as solutions for complications, including coronary heart disease and dilation of the aortic arch and Stanford type B aortic dissection. Computed tomography and intraoperative images showed typical ascending aortic aneurysms (Figure 1(a)). For example, in Figure 1 an obvious ascending aortic aneurysm was shown with a diameter of over 60 mm. Derangement of VSMCs and dissection of elastic lamellae were observed by elastin staining in patient samples (Figure 1(b)). Through confocal fluorescence microscopy analysis of TUNEL and *α*-SMA double staining, the increase of VSMC apoptosis in patients with ascending aortic aneurysms was clear compared with the normal control samples (Figure 1(c)). Although there were also some apoptotic cells in the aortic walls of normal human ascending aortas, they were rarely VSMCs (Figure 1(c)). Based on counting the number of apoptotic VSMCs in different views, VSMC apoptosis in the aortic walls of patients with ascending aortic aneurysms was found to be significantly higher than that of the normal human control samples (Figure 1(d), *p* = 0.0014). We also detected the apoptosis of VSMCs in patients with ascending aortic aneurysms by immunohistochemical staining of cleaved caspase-3 and Bcl-2 (Figure 1(e)). Immunohistochemistry revealed that cleaved caspase-3 staining was strong and Bcl-2 staining was weak in the ascending aortic walls of ascending aortic aneurysms relative to normal, both of which suggested increased apoptosis in the aortic walls of patients with ascending aortic aneurysms.

### 3.2. YAP Expression Was Reduced in the Aortic Walls of Patients with Ascending Aortic Aneurysms

**Figure 2:**
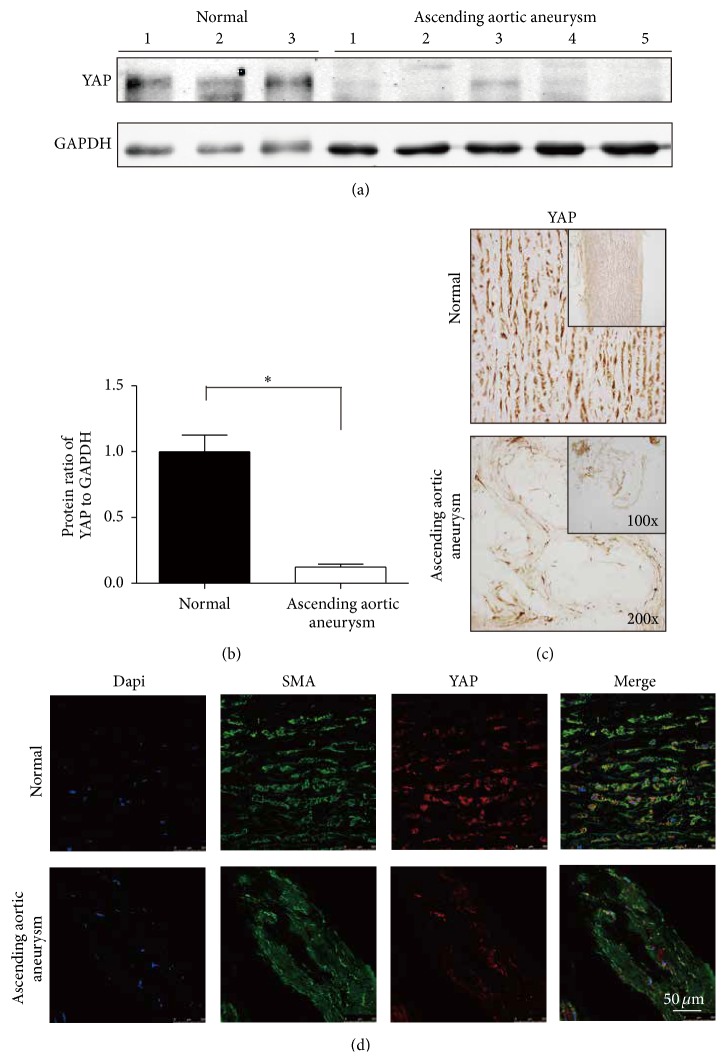
(a, b) Western blotting showed that expression of YAP decreased significantly in the ascending aortic walls of patients with ascending aortic aneurysms compared to that of normal controls (^*∗∗*^ *p* = 0.0047). (c) Immunohistochemistry showed that YAP was weak in the ascending aortic walls of ascending aortic aneurysms relative to normal. (d) Confocal fluorescence microscopy showed that YAP and *α*-SMA double-staining cells were reduced in ascending aortic aneurysms.

To further assess the expression of YAP, we performed Western blotting and immunohistochemical staining to evaluate the abundance of the protein. We found a significant reduction of YAP at the protein level in patients with ascending aortic aneurysms. Western blotting revealed that the expression of YAP decreased in the ascending aortic walls of patients with ascending aortic aneurysms compared with that of normal controls (Figure 2(a)), and the results analyzed using the Odyssey infrared imaging system revealed that this difference was significant (Figure 2(b), *p* = 0.0047). Furthermore, immunohistochemistry showed that YAP was weak in the aortic walls of ascending aortic aneurysms relative to normal, which also suggested the downregulation of YAP in the aortic walls of ascending aortic aneurysms (Figure 2(c)). The confocal fluorescence microscopy analysis of YAP and *α*-SMA double staining showed that the expression of YAP was reduced in VSMCs from the aortic walls of patients with ascending aortic aneurysms (Figure 2(d)), which revealed the downregulation of YAP in VSMCs of ascending aortic aneurysms.

## 4. Discussion

Our findings regarding the changes of the aortic wall are consistent with those reported by previous studies [18–20] that also suggest the significance of VMSC apoptosis in the pathology of aortic medial degeneration. Given the difficulty of obtaining clinical ascending aortic aneurysm samples, the pathogenesis has not yet been fully elucidated, and most of the current hypotheses come from the research of abdominal aortic aneurysms [21, 22]. However, due to the significant differences between ascending aortic aneurysms and abdominal aortic aneurysms in terms of epidemiology, embryonic origin, genetic development, hemodynamics, and pathology, these hypotheses of etiology speculated from the research results of abdominal aortic aneurysms cannot completely clarify the pathogenesis of ascending aortic aneurysms [23]. In recent years, with the development of various experimental techniques, the study of the etiology of ascending aortic aneurysms has gradually increased. The existing research results show that the occurrence of ascending aortic aneurysms is a result of the interaction of the ECM and VSMCs: Changes in the ECM will induce the apoptosis of VSMCs, influence the function of the aortic wall, reduce the generation of elastic fibers, and then cause the expansion of the ascending aorta [24–26]. The occurrence of many aortic diseases is closely related to the apoptosis of aortic VSMCs [14–16, 27]. In animal aortic injury models, the apoptosis of aortic VSMCs is observed [28, 29]. However, there is no known mechanism of VSMC apoptosis in the aortic walls of patients with ascending aortic aneurysms.

The wall of the aorta contains three layers. The media is made up of collagen, VSMCs, and roughly 50 elastic laminas, which can be highly stretched [30]. The etiology of ascending aortic aneurysms is mainly histopathology and genetic factors, which suggest that preexisting weakness of the aortic media is the basis of the occurrence of ascending aortic aneurysms [1, 31]. Media degeneration refers to cystic degeneration or necrosis of elastic fibers, including rupture of the elastic lamina and apoptosis of VSMCs [32, 33]. Media degeneration also generates ECM mechanical stress. Moreover, apoptosis of VSMCs could be induced by cyclic stretch, which is thought to be a simulation of mechanical stress [17]. Media degeneration, including rupture of the elastic lamina and apoptosis of VSMCs, has been reported in human ascending aortic aneurysms [31, 32]. Consistent with this, aortic wall samples of human ascending aortic aneurysms displayed disrupted elastic lamellae, which indicates medial degeneration in our study. Upon costaining of TUNEL with *α*-SMA, the increase of VSMC apoptosis in the aortic walls of patients with ascending aortic aneurysms is clear compared with the normal control samples. We also detected apoptosis of VSMCs by performing staining of cleaved caspase-3, and we found results similar to those obtained by the costaining of TUNEL with *α*-SMA.

Some members of the Hippo-YAP pathway have been reported to have the ability to induce apoptosis in rat aortic VSMCs [34]. In this study, we observed a similar phenomenon that downregulated YAP was accompanied by VSMC apoptosis and was associated with ECM disorders in ascending aortic aneurysms. The above result resembles those reported by previous studies: Because mechanical stress caused by differences in the ECM is crucial to regulating the expression of YAP [13], YAP will be inhibited when cells are cultured on soft matrix [35]. Clearly, disrupted elastic lamellae with variable width in STAAD soften the ECM, which might induce the downregulation of YAP.

There are several limitations of the present study. Most obviously, the results are only the description of phenomenon in the ascending aortic aneurysm. However, given the precious value of the aortic wall samples of human ascending aortic aneurysms, this is the most realistic representation of ascending aortic aneurysms. Further studies to elucidate the mechanisms of ascending aortic aneurysms* in vivo* and* in vitro* will be continued in the future; however, the results of this paper provide the basis for such mechanistic studies. Furthermore, due to the low numbers of samples included in the present study, we may have overestimated the effect of the downregulation of YAP in VSMCs. Most notably, the role of YAP in the apoptosis of VSMCs of the media in ascending aortic aneurysms as observed in this study needs to be examined further.

## 5. Conclusions

Our study is the first to show that the downregulation of YAP in VSMCs is closely associated with ECM disorders of the media in ascending aortic aneurysms. The downregulated YAP accompanied by the alteration in mechanical stress caused by ECM disorders in the aortic wall is essential for aortic structure and function, and apoptosis of VSMCs following the downregulation of YAP may contribute to the pathogenesis of ascending aortic aneurysms.

## Figures and Tables

**Table 1:** Ascending aortic aneurysm patients' information related to the aortic tissue used in the study.

Number	Age	Gender	Diameter of ascending aorta (mm)	Diameter of aortic sinus (mm)	Aortic insufficient	Aortic bicuspid	Complications	Surgery
1	65	Male	55	55	Moderate	No	Stanford type B aortic dissection	Ascending aorta replacement + total arch replacement by a tetrafurcate graft and stented elephant trunk implantation
2	70	Male	52	52	Mild	No	Coronary heart disease and dilation of aortic arch	Ascending aorta replacement + partial aortic arch + CABG
3	59	Male	53	50	Severe	No	None	Bentall
4	74	Male	69	41	Mild	No	Coronary heart disease and dilation of aortic arch	Ascending aorta replacement + partial aortic arch + CABG
5	52	Female	59	40	Moderate	No	Dilation of aortic arch	Bentall + partial aortic arch replacement
6	60	Female	51	37	Severe	No	Mitral regurgitation	Bentall + MVR
7	54	Female	57	36	Severe	Yes	None	Bentall
8	54	Male	50	35	Moderate	Yes	Stanford type B aortic dissection	Ascending aorta replacement + total arch replacement by a tetrafurcate graft and stented elephant trunk implantation

CABG: Coronary Artery Bypass Graft; MVR: Mitral Valve Replacement.
